# Screening of Antiglaucoma, Antidiabetic, Anti-Alzheimer, and Antioxidant Activities of *Astragalus alopecurus* Pall—Analysis of Phenolics Profiles by LC-MS/MS

**DOI:** 10.3390/ph16050659

**Published:** 2023-04-28

**Authors:** Leyla Güven, Adem Erturk, Fatma Demirkaya Miloğlu, Saleh Alwasel, İlhami Gulcin

**Affiliations:** 1 Department of Pharmaceutical Botany, Faculty of Pharmacy, Ataturk University, 25240 Erzurum, Turkey; 2 Department of Pharmacy Services, Hınıs Vocational School, Ataturk University, 25600 Erzurum, Turkey; a.erturk@atauni.edu.tr; 3 Department of Analytical Chemistry, Faculty of Pharmacy, Ataturk University, 25240 Erzurum, Turkey; fdkaya@atauni.edu.tr; 4 Department of Zoology, College of Science, King Saud University, Riyadh 11362, Saudi Arabia; salwasel@ksu.edu.sa; 5 Department of Chemistry, Faculty of Science, Ataturk University, 25240 Erzurum, Turkey

**Keywords:** acetylcholinesterase, antioxidant activity, astragalus, carbonic anhydrase, α-amylase, α-glycosidase, LC-MS/MS

## Abstract

Astragalus species are traditionally used for diabetes, ulcers, leukemia, wounds, stomachaches, sore throats, abdominal pain, and toothaches. Although the preventive effects of Astragalus species against diseases are known, there is no record of the therapeutic effects of *Astragalus alopecurus*. In this study, we aimed to evaluate the in vitro antiglaucoma, antidiabetic, anti-Alzheimer’s disease, and antioxidant activities of the methanolic (MEAA) and water (WEAA) extracts of the aerial part of *A. alopecurus*. Additionally, its phenolic compound profiles were analyzed by liquid chromatography–tandem mass spectrometry (LC–MS/MS). MEAA and WEAA were evaluated for their inhibition ability on α-glycosidase, α-amylase, acetylcholinesterase (AChE), and human carbonic anhydrase II (hCA II) enzymes. The phenolic compounds of MEAA were analyzed by LC-MS/MS. Furthermore, total phenolic and flavonoid contents were determined. In this context, the antioxidant activity was evaluated by 1,1-diphenyl-2-picrylhydrazyl (DPPH), 2,2′-azino-bis(3-ethylbenzothiazoline-6-sulfonic acid) (ABTS), N,N-dimethyl-p-phenylene diamine (DMPD), ferric reducing antioxidant power (FRAP), cupric ions (Cu^2+^) reducing antioxidant capacity (CUPRAC), ferric ions (Fe^3+^) reducing, and ferrous ions (Fe^2+^) chelating methods. MEAA and WEAA had IC_50_ values of 9.07 and 2.24 μg/mL for α-glycosidase, 693.15 and 346.58 μg/mL for α-amylase, 1.99 and 2.45 μg/mL for AChE, and 147.7 and 171.7 μg/mL for hCA II. While the total phenolic amounts in MEAA and WEAA were 16.00 and 18.50 μg gallic acid equivalent (GAE)/mg extract, the total flavonoid contents in both extracts were calculated as 66.23 and 33.115 μg quercetin equivalent (QE)/mg, respectively. MEAA and WEAA showed, respectively, variable activities on DPPH radical scavenging (IC_50_: 99.02 and 115.53 μg/mL), ABTS radical scavenging (IC_50_: 32.21 and 30.22 µg/mL), DMPD radical scavenging (IC_50_: 231.05 and 65.22 μg/mL), and Fe^2+^ chelating (IC_50_: 46.21 and 33.01 μg/mL). MEAA and WEAA reducing abilities were, respectively, Fe^3+^ reducing (λ_700_: 0.308 and 0.284), FRAP (λ_593_: 0.284 and 0.284), and CUPRAC (λ_450_: 0.163 and 0.137). A total of 35 phenolics were scanned, and 10 phenolic compounds were determined by LC-MS/MS analysis. LC-MS/MS revealed that MEAA mainly contained isorhamnetin, fumaric acid, and rosmarinic acid derivatives. This is the first report indicating that MEAA and WEAA have α-glycosidase, α-amylase, AChE, hCA II inhibition abilities, and antioxidant activities. These results demonstrate the potential of Astragalus species through antioxidant properties and enzyme inhibitor ability traditionally used in medicine. This work provides the foundation for further research into the establishment of novel therapeutics for diabetes, glaucoma, and Alzheimer’s disease.

## 1. Introduction

Medicinal plants are the most important source of natural medicines used in traditional and modern treatment methods. For this reason, plants used for medicinal purposes have been widely used in the treatment of many diseases for a long time [1]. The use of medicinal plants in our daily lives is accepted as a complementary and alternative treatment along with other treatments [2]. It is known that most diseases occurring in metabolism are associated with the abnormal formation of free radicals. However, it should not be forgotten that free radicals are an important part of aerobic life and metabolic activities. Because these reactive intermediates are extremely indispensable for most biochemical processes [3], it is known that reactive oxygen species (ROS) and free radicals are associated with the etiology of many diseases, especially cancer, Parkinson’s disease, Alzheimer’s disease (AD), diabetes, and cardiovascular diseases [4,5]. Medicinal plants have antioxidant effects and contain many phenolic compounds that protect living organisms against free radical-induced damage and diseases [6,7].

Antioxidants protect or completely prevent cellular components and biomolecules from oxidative damage caused by free radicals and reactive oxygen species (ROS) [8]. Antioxidants can delay, reduce, or completely eliminate the oxidation process and the formation of free radicals. Antioxidants scavenge free radicals and ROS by donating electrons. Detection of antioxidant activities in plants used for medicinal purposes and identification of phenolic compounds in the plant are important for obtaining new antioxidant molecules [9]. Additionally, iron accumulation and oxidative damage are major risk factors for neurological illnesses, cancer, and other diseases [10]. Antioxidants perform these functions by terminating radical chain reactions, removing radical intermediates, or inhibiting oxidation reactions [11]. The antioxidant defense system in humans is rich in substances that can prevent the formation of free radicals or their possible damage. However, in cases where antioxidant defense is insufficient, it should be supplemented externally with a diet rich in polyphenolic content [12]. In this context, plants are very rich in secondary metabolites that remove the oxidative damage of free radicals and have antioxidant effects [13]. Polyphenols, as secondary metabolites, are the main dietary phenolics, including phenolic acid and flavonoids. Of these, flavonoids are the most studied group of polyphenols [14,15]. They exhibit antioxidant potential by preventing the decomposition of hydroperoxides into free radicals and neutralizing ROS and free radicals. Here, the Astragalus family is extremely rich in these secondary metabolites and is frequently used medicinally [16,17].

Astragalus L. (Fabaceae) has 3000 species in the world, approximately 480 taxa, and 202 endemic species in Turkey, and the endemism rate is 42%. Astragalus species are traditionally used in the treatment of diabetes, ulcers, leukemia, wounds, stomachaches, sore throats, abdominal pain, and toothaches [18,19,20]. Furthermore, it has also been used in the modulation of the immune system [21] and the protection of the heart [22]. The root and aerial parts of the plant are generally used in the form of decoction, brewing, bathing, porridge, chewing, infusion, and powder [23]. Some pharmacological studies on Astragalus species indicate that they exhibit anti-inflammatory [24], neuroprotective [25], immunomodulatory [26], antioxidative, antidiabetic, anticholinergic [24], cardiotonic, hypocholesterolemic, anti-depressive, antiblastic [21], anticancer [27], anti-aging [28], hepatoprotective [29], and antiviral activities [30]. Astragalus species primarily contain polysaccharides in the glucan structure [31]. In addition, they contain flavonoids such as hyperoside, apigenin, kaempferol, naringenin [18], cycloartane and lanostane type saponins, phenolic acids [32], proanthocyanidins, alkaloids, and tannins [33]. In the study by Agzamova and Isaev, it was reported that cycloalpigenin D, cycloalpioside D, cycloalpigenin D, and cycloalpigenin 3,7-diacetate triterpenoids were isolated from *A. alopecurus* [34]. Ghahari et al. reported that the major components in the GC-MS/MS analysis of *A. alopecurus* fruit essential oils were 18.41% α-pinene, 12.84% humulene epoxide II, and 11.81% α-humulene. Essential oils of *A. alopecurus* fruits showed antimicrobial activity against *Staphylococcus aureus* (MIC: 50 μg/mL) and *Pyricularia oryzae* (MIC: 12.5 μg/mL). The amounts of phenolic, flavonoid, and alkaloid substances in the fruits of the plant are 53.61 mg/g, 115.64 mg/g, and 0.11 mg/g, respectively [35].

Antioxidants are known to protect cellular biocomponents from damage caused by free radicals. In this context, they do so by slowing down or completely preventing the oxidation of biomolecules [36]. Antioxidants have properties such as terminating radical chain reactions, removing radical intermediates, and inhibiting oxidation reactions by oxidizing themselves. Antioxidant-effective plants and their phytochemical compounds are suitable for the treatment of diabetes, Alzheimer’s disease (AD), glaucoma, Parkinson’s disease, and vitiligo, according to some studies [37]. They treat or prevent these diseases by inhibiting enzymes such as α-amylase, α-glycosidase, butyrylcholinesterase (BChE), acetylcholinesterase (AChE), and carbonic anhydrase (CA). Therefore, antioxidants can help treat the aforementioned diseases. It is also known that there is a relationship between diabetes and AD [27]. Due to the different side effects of synthetic drugs, interest in natural products is increasing. Phytochemical components in plants can facilitate treatment with a synergistic effect [31,38]. AD is a multifaceted neurodegenerative disease that arises and is characterized by changes in memory, behavior, reasoning, emotions, and abstract thinking [39]. The cholinergic hypothesis, which best explains the symptoms and pathophysiology of the disease, is accepted as one of the main hypotheses. According to this hypothesis, cholinergic neurotransmission is thought to have a vital role in the neural functions of AD patients [40]. The cholinergic hypothesis is the most widely accepted therapeutic tool for improving cognitive functions in AD patients. According to this hypothesis, treatments that inhibit AChE play an essential role in the development of AD. AChE inhibitors are considered one of the most symptomatic treatments for AD, which is a neurodegenerative, irreversible, and progressive disorder [41]. AChE, as a serine hydrolase, is an essential enzyme located at cholinergic synapses and has a very important role in cognition and memory. Inhibition of this enzyme has been recognized as a therapeutic strategy for AD as well as other common diseases such as myasthenia gravis, dementia, Parkinson’s disease, and glaucoma. The use of Donepezil, Galantamine, and Rivastigmine has been limited due to their side effects, including hepatotoxicity, gastrointestinal disorders, and diarrhea. Therefore, the demand for compounds with no side effects and a natural origin is increasing [42,43].

CAs are a very large family of metalloenzymes containing Zn^2+^ ions that catalyze the reversible conversion of carbon dioxide (CO_2_) and water to proton (H^+^) and bicarbonate (HCO_3_^−^) in all living organisms [44]. To date, carbonic anhydrases (α, β, γ, δ, ξ, η, θ and t-CAs) have been broadly classified into eight different genetic families [45]. The α-CA family includes sixteen isoenzymes with different kinetic properties, subcellular locations, and inhibitor profiles. Of these, the hCA II isoform is found in the cytosol and is the most dominant isozyme [46]. In addition to its high concentration in different regions of the eye, such as the retina and lens, hCA II is also found in the kidney, pancreas, brain, gastric mucosa, skeletal muscle, red blood cells (RBCs), testicles, lungs, and osteoclast [47]. There is an established and clear relationship between glaucoma and the human CA II isoenzyme. It is known that hCA II isoenzyme found in ciliary epithelial cells in the ciliary body reduces aqueous humor secretion and thus lowers intraocular pressure (IOP) [48]. The most commonly known mechanism of action of anti-glaucoma agents is inhibition of the hCA II isozyme, which reduces HCO_3_^−^ and H^+^ production, resulting in a decrease in high IOP [49]. Thus, it is seen that hCA II plays an important and quite effective role in the IOP regulation. Today, it is known that around 67 million people worldwide have glaucoma, which is the most important factor known to cause blindness. This number is expected to affect approximately 112 million people by 2040. In this context, the development and discovery of highly potent antiglaucoma agents with fewer side effects is very important for ophthalmic drug design [50]. Dorzolamide, an inhibitor of carbonic anhydrase (CA) isoenzymes, has been used in the treatment of glaucoma since it was approved in 1995. In the treatment of glaucoma, it is necessary to reduce ocular hypertension to lower the IOP. For this purpose, β-blockers, topical prostaglandins, CA inhibitors (CAIs), or their combinations are the most commonly applied methods to date [47].

Diabetes mellitus (DM) is a common and multifactorial metabolic disease characterized by hyperglycemia due to insulin deficiency or insulin resistance [51]. According to a classification made by the American Diabetes Association (ADA), diabetes types are classified into four general categories: The first is insulin-dependent Type-1 DM (T1DM), the second is insulin-dependent Type-2 DM (T2DM), the third is neonatal diabetes, and the last is gestational diabetes [52]. Non-insulin-dependent T2DM is the most common type of diabetes and accounts for approximately 90–95% of all diabetes cases [53]. According to the World Health Organization’s data, T2DM continues to be the most common and fastest rising health problem in developing countries [54]. In T2DM, long-term high glucose levels trigger the deterioration of cellular functions, especially inflammatory and oxidative stress, leading to the development of serious chronic diabetic complications such as neuropathy, cataracts, and atherosclerosis [55]. One of the therapeutic approaches to reducing hyperglycemia is inhibition of α-glycosidase and α-amylase, which break down α-1,4 glycosidic bonds from the non-reducing ends of oligosaccharides and polysaccharides, allowing them to be absorbed by the small intestine and enter the bloodstream [56]. α-Glycosidase inhibitors competitively inhibit intestinal α-glycosidase, thereby delaying or decreasing carbohydrate absorption in the small intestine. The use of α-glycosidase inhibitors is widely used to treat diabetes, particularly T2DM. Therefore, the most important features of the ideal antidiabetic agent are that it is of natural origin, has a hypoglycemic effect, and has the ability to prevent long-term diabetic complications [53,57].

In this study, we aimed to determine the antiglaucoma, antidiabetic, anticholinergic, antioxidant, and other activities of methanol (MEAA) and water (WEAA) extracts of the aerial parts of *A. alopecurus*. For this purpose, the possible inhibitory effects of MEAA and WEAA toward AChE, hCA II, α-amylase, and α-glycosidase enzymes were determined. Another goal of this study was to investigate the antioxidant ability of both extracts with different bioanalytical methods, including potassium ferricyanide reduction, Fe^3+^-2,3,5-Triphenyltetrazolium chloride (TPTZ) reduction (FRAP), copper ion (Cu^2+^) reducing capacity (CUPRAC), DPPH˙, and ABTS^+^ scavenging and metal chelating. Additionally, the total phenolic and flavonoid contents of MEAA and WEAA extracts were also defined. The polyphenolic analysis of MEAA was quantitatively determined by LC-MS/MS. Another goal of this study was to identify the possible inhibitory effects of MEAA and WEAA on AChE, hCA II, α-amylase, and α-glycosidase metabolic enzymes.

## 2. Results

### 2.1. Total Phenolic and Flavonoid Contents

Total phenolic contents in MEAA and WEAA were calculated using standard gallic acid calibration curves by the Folin-Ciocalteu reagent (r^2^: 0.9983) [58]. The quantities of phenolics in MEAA and WEAA were determined using the standard gallic acid equation and found to be 16.00 and 18.50 μg GAE/mg extract, respectively. Furthermore, flavonoids are one of the most abundant secondary metabolites in medicinal plants. It was determined that *A. alopecurus* contains 66.23 and 33.115 μg QE/mg flavonoid in methanol and water extracts, respectively.

### 2.2. Polyphenolic Analysis by LC-MS/MS

**Figure 1:**
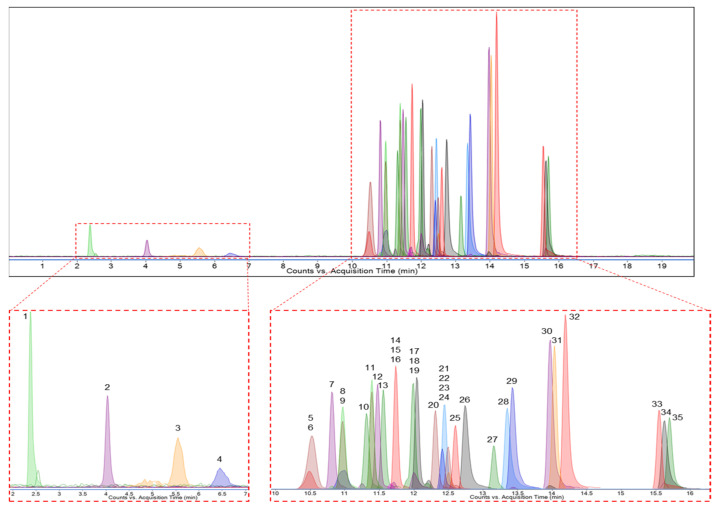
Multiple reaction monitoring (MRM) chromatogram of standard phenolic compounds. 1: Quinic acid, 2: Fumaric acid, 3: Gallic acid, 4: Pyrogallol, 5: Keracyanin Chloride, 6: Cyanidin-3-*O*-glucoside, 7: Chlorogenic acid, 8: Catechin, 9: Peonidin-3-*O*-glucoside, 10: 4-Hydroxy benzoic acid, 11: Epicatechin, 12: Epigallocatechin gallate, 13: Caffeic acid, 14: Vanillic acid, 15: Syringic acid, 16: Vitexin, 17: Naringin, 18: Ellagic acid, 19: Hesperidin, 20: *p*-Coumaric acid, 21: Sinapic acid, 22: Taxifolin, 23: Ferulic acid, 24: Rosmarinic acid, 25: Vanillin, 26: Myricetin, 27: Resveratrol, 28: Luteolin, 29: Quercetin, 30: Apigenin, 31: Naringenin, 32: Isorhamnetin, 33: Chrysin, 34: Galangin, and 35: Curcumin.

**Figure 2:**
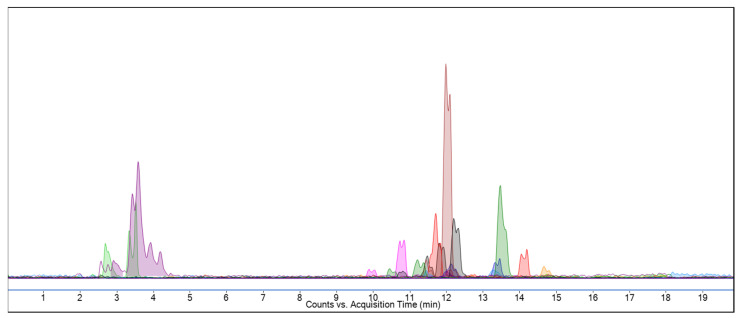
Multiple reaction monitoring (MRM) chromatogram of the methanolic extract (MEAA) of the aerial part of *A. alopecurus* analyzed by the LC-MS/MS method.

Phenolic compounds are bioactive secondary metabolites found in plants with potential beneficial effects on human health [59]. The LC-MS/MS method is a widely used technique for the analysis of phenolic compounds found in plants. Product ions are produced by tandem mass spectrometry, which allows the characterization of compounds in a given sample. LC-MS/MS is both a powerful and accurate technique for the qualitative and quantitative analysis of phenolic compounds due to its method versatility [60]. In this study, 35 of all known phenolic compounds that have been characterized using the developed method were searched for in MEAA extracts. Figure 1 and Figure 2 show LC-MS/MS chromatograms of 35 standard phenolic compounds and MEAA extract, respectively.

The analytical method was validated for linearity, intra- and inter-day precision, accuracy, limit of detection (LOD), and limit of quantification (LOQ). Linearity was demonstrated by linear calibration curves obtained by plotting the peak area versus the concentration of each phenolic compound. The method showed good linearity in the concentration range of 100–2000 μg/L for 30 phenolic compounds, 250–2000 μg/L for 4-OH-benzoic acid and caffeic acid, and 100–1500 μg/L for apigenin, naringenin, and galangin. Intra- and inter-day precision and accuracy were determined by analyzing three replicates of quality control (QC) samples for each phenolic compound on a single day and on three separate days, respectively. The QC samples were the 100, 750, and 1500 μg/L concentrations, except for 4-OH-benzoic acid and caffeic acid (250, 750, and 1500 μg/L) for all phenolic compounds. Precision and accuracy were defined as relative standard deviation (RSD) and relative error (RE), respectively. According to the analyzed results of each phenolic compound, the intra- and inter-day RSD% and RE% were less than 2.48% and ±1.52 at the QC concentrations, respectively. The sensitivity of the method was determined with LOD and LOQ defined as 3SDy/x and 10SDy/x, respectively, where SDy is the standard deviation of the y-intercepts and x is the slope of the calibration curves for each phenolic compound. The highest LOD/LOQ values were obtained for epigallocatechin gallate (1.98/6.6 μg/L) and the lowest for naringin (0.33/1.08 μg/L). The LOD/LOQ values for all other phenolic compounds were within these ranges.

The phenolic compounds in MEAA were determined according to the MS spectra and the retention times of the reference standards. Each sample had two measurements. According to the results, 10 phenolic compounds: fumaric acid, chlorogenic acid, 4-OH-benzoic acid, ellagic acid, p-coumaric acid, rosmarinic acid, luteolin, quercetin, naringenin, and isorhamnetin were identified in MEAA in Table 1. Quantitative analysis of the identified phenolic compounds in MEAA was determined by using calibration curves with seven concentration levels for each analyte, and each level was analyzed in triplicate. When they were ordered from the highest amount to the lowest, isorhamnetin (1489.11 µg/L), fumaric acid (855.07 µg/L), rosmarinic acid (64.54 µg/L), 4-OH-benzoic acid (57.23 µg/L), ellagic acid (48.54 µg/L), chlorogenic acid (40.92 µg/L), p-coumaric acid (32.25 µg/L), quercetin (10.88 µg/L), luteolin (5.50 µg/L), and naringenin (1.44 µg/L) were found in the MEAA by LC-MS/MS analysis (Table 1).

### 2.3. Antioxidant Results

**Figure 3:**
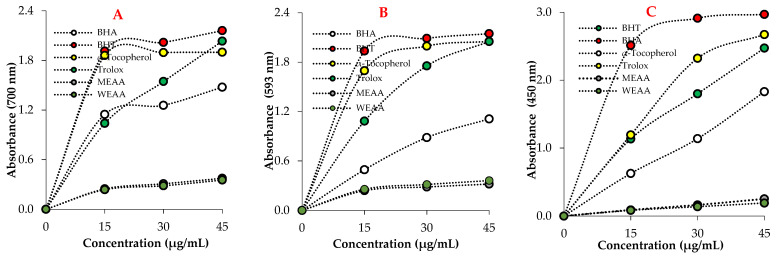
Reductive potentials of methanolic (MEAA) and water (WEAA) extracts of the aerial part of *A. alopecurus* and positive controls. (**A**) Ferrous ions (Fe^3+^) reducing ability; (**B**) Ferrous ions (Fe^3+^) -2,3,5-triphenyltetrazolium chloride (TPTZ) reducing ability; and (**C**) Cupric ions (Cu^2+^) reducing ability.

The reducing activity of MEAA and WEAA was evaluated by measuring their ability to reduce Fe^3+^ to Fe^2+^. Various electron-donating functional groups, such as -OH, -SH, and -COOH belonging to the compounds found in plant extracts, are of great importance to the reducing capacity [61]. As shown in Figure 3, MEAA and WEAA show inferior reducing power compared to the standards when utilizing the potassium ferricyanide reduction technique. The Oyaizu method [41] was used to explore the Fe^3+^-Fe^2+^ transition in order to determine the reductive capacity of MEAA and WEAA, which displayed high reducing activity at various concentrations (15–45 μg/mL). With rising sample concentrations, the reducing power of Trolox, BHA, BHT, α-tocopherol, MEAA, and WEAA continuously rose. The sequence of the standard compounds and extracts reducing abilities was BHT (λ_700_: 2.018) > α-Tocopherol (λ_700_: 1.895) > Trolox (λ_700_: 1.545) > BHA (λ_700_: 1.257) > MEAA (λ_700_: 0.308) > WEAA (λ_700_: 0.284). The outcomes show that plant extracts have the ability to donate electrons to stable compounds, neutralizing free radicals (Figure 3A and Table 2).

**Figure 4:**
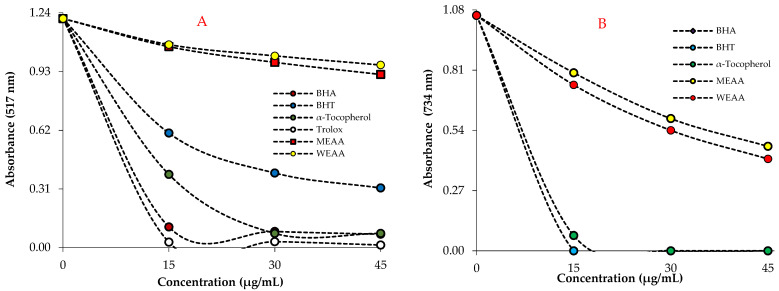

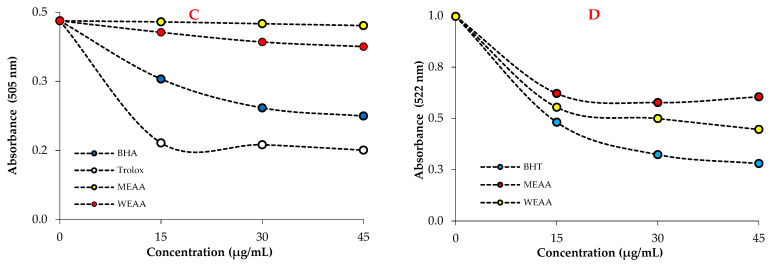
Radical scavenging and metal chelating effects of methanolic (MEAA) and water (WEAA) extracts of the aerial part of *A. alopecurus* and positive controls. (**A**) 1,1-diphenyl-2-picrylhydrazyl radicals (DPPH^•^) scavenging ability; (**B**) 2,2′-azino-bis(3-ethylbenzothiazoline-6-sulfonic acid) (ABTS^•+^) scavenging ability, (**C**) N,N-dimethyl-p-phenylene diamine (DMPD^•+^) scavenging ability; and (**D**) Ferrous ions (Fe^2+^) chelating ability.

According to the results of the reduction capacity of ferric ions (FRAP), the reducing capacity of MEAA and WEAA increased with the increase in concentration. In addition, when the reducing capacity of the studied extracts and standard antioxidants was compared with absorbances at 30 μg/mL, BHT (λ_593_: 2.089) > α-Tocopherol (λ_593_: 1.995) > Trolox (λ_593_: 1.755) > BHA (λ_593_: 0.884) > WEAA (λ_593_: 0.284) = MEAA (λ_593_: 0.284) (Table 2 and Figure 3B). As shown in Figure 4, the activity of each reducing ability increased with increasing concentrations (15–45 μg/mL).

According to the results of the reducing capacity of cupric ions (Cu^2+^) (CUPRAC), the reducing capacities of MEAA and WEAA increased depending on the concentration increase. The Cu^2+^-reducing capacity of MEAA and WEAA was determined by measuring the absorbance of solutions at different concentrations (15–45 μg/mL) at 450 nm. When the absorbances of MEAA, WEAA, and standards were compared at 30 μg/mL, the following order was found: BHT (λ_450_: 2.912) > Trolox (λ_450_: 2.323) > BHA (λ_450_: 1.800) > α-Tocopherol (λ_450_: 1.139) > MEAA (λ_450_: 0.163) > WEAA (λ_450_: 0.137) (Table 2, Figure 3C).

The IC_50_ values of DPPH scavenging of both extracts and standard antioxidants decreased in the following order: WEAA (115.53 µg/mL; r^2^: 0.9934) > MEAA (99.02 µg/mL; r^2^: 0.9977) > BHT (21.00 µg/mL; r^2^: 0.9668) > *α*-tocopherol (9.63 µg/mL; r^2^: 0.9947) > BHA (9.00 µg/mL; r^2^: 0.9399) > Trolox (5.92 µg/mL; r^2^: 0.9770) (Table 3 and Figure 4A).

Both *A. alopecurus* extracts exhibited an effective ABTS radical scavenging profile (*p* > 0.001). IC_50_ values for MEAA and WEAA in this assay were determined as 32.21 μg/mL (r^2^: 0.9987) and 30.22 μg/mL (r^2^: 0.9976). Additionally, IC_50_ values were found to be 7.71 μg/mL (r^2^: 0.9330) for BHA, 7.71 μg/mL (r^2^: 0.9330) for BHT, 8.10 μg/mL (r^2^: 0.9550) for α-tocopherol, and 7.71 μg/mL (r^2^: 0.9330) for Trolox as a water-soluble analogue of α-tocopherol (Table 3 and Figure 4B). Radical scavenging ability is frequently used for screening the antioxidant properties of plant extracts. In this study, the third evaluated radical scavenging assay is DMPD^•+^ removing activity. The DMPD^•+^ scavenging assay had a very stable endpoint that was comparable to the ABTS^•+^ scavenging assay. As shown in Table 3, both MEAA and WEAA extracts had effective DMPD radical scavenging in a concentration-dependent manner (15–45 μg/mL). The IC_50_ values of MEAA and WEAA were calculated as 231.05 μg/mL (r^2^: 0.9967) and 65.22 μg/mL (r^2^: 0.9987), respectively, whereas the IC_50_ values were found to be 31.43 μg/mL (r^2^: 0.9993) for BHA and 14.38 μg/mL (r^2^: 0.9349) for Trolox (Table 3 and Figure 4C). These medicinal plants contain a broad spectrum of biologically active substances [42]. When metal chelating activity is evaluated, IC_50_ values for MEAA, WEAA, and the reference standard agent BHT were determined as 46.21 μg/mL (r^2^: 0.9717), 33.01 μg/mL (r^2^: 0.9601), and 21.66 μg/mL (r^2^: 0.9908), respectively (Table 3, Figure 4D).

### 2.4. Enzyme Inhibition Results

The antidiabetic activity of MEAA and WEAA was assessed using α-amylase and α-glycosidase inhibition assays in the study. The findings are shown in Table 4. MEAA and WEAA had IC_50_ values for α-glycosidase of 9.07 μg/mL (r^2^: 0.9775) and 2.24 μg/mL (r^2^: 0.9155), respectively. This value was determined to be 693.15 μg/mL (r^2^: 0.9677) and 346.58 μg/mL (r^2^: 0.9856), respectively, for α-amylase (Table 4). Acarbose, a common antidiabetic medication, was utilized as a standard reference [62]. According to the findings, methanol and water extracts inhibited both enzymes at a level proximate to that of the standard antidiabetic drug. The results in Table 4 show that aqueous extract inhibited the α-glycosidase and α-amylase enzymes better than methanol extract. In future studies, isolation studies on these two extracts will allow for obtaining the pure substances responsible for the effect.

The inhibitory effects of MEAA and WEAA on the AChE enzymes associated with AD in different doses were examined, and IC_50_ values were obtained. The results in Table 4 show that methanol extract inhibits the AChE enzyme better than aqueous extract. In our study, the results of the effects of MEAA and WEAA on AChE inhibition were evaluated. According to the findings, it was determined that MEAA and WEAA had IC_50_ values of 1.99 μg/mL (r^2^: 0.9923) and 2.45 μg/mL (r^2^: 0.9930) for AChE, respectively. In addition, plant extracts were found to have a lower inhibitory activity than standard inhibitory tacrine (IC_50_ 0.0246 μg/mL; r^2^:0.9706). In the current study, MEAA and WEAA inhibited hCA II isoenzyme with IC_50_ values of 147.7 μg/mL (r^2^: 0.9804) and 171.7 μg/mL (r^2^: 0.9671), respectively.

Additionally, cytosolic and dominant CA II isoenzymes are associated with a number of disorders, such as glaucoma, osteoporosis, and renal tubular acidosis. The IC_50_ values of MEAA and WEAA towards CA isoenzymes were found to be 147.7 μg/mL (r^2^: 0.9804) and 171.7 μg/mL (r^2^: 0.9671), respectively (Table 4). On the other hand, acetazolamide (AZA), which is clinically used as a control for the inhibition of CA isoenzymes, displayed an IC_50_ value of 8.37 µg/mL (r^2^: 0.9825).

## 3. Discussion

All polyphenolic compounds identified in the present study are known as bioactive components with protective effects against diseases such as inflammation, autoimmune disease, neurodegenerative disease, and cancer [63]. A previous study on Astragalus species identified chlorogenic acid, epicatechin, catechin hydrated, rutin, quercetin (293.5 µg/L), kaempferol, syringic acid (735.18 µg/L), cinnamic acid (558 µg/L), and ferulic acid (1123.9 µg/L) [64]. In the other study by Krasteva and Nikolov [65], nine flavonoids (vitexin, orientin, eriodictyol-7-*O*-glucoside, isorhamnetin-3-*O*-glucoside, kaempferol, isorhamnetin, isorhamnetin-3-*O*-rutinoside, quercetin, and quercetin-3-*O*-glucoside/galactoside/rutinoside) were identified. The LC-MS/MS method also identified phenolic compounds containing rosmarinic, protocatechuic, chlorogenic acids, 4-hydroxybenzoic, hyperoside, and hesperidin in the ethanol extract of *A. armatus* [66]. In addition, ferulic, p-coumaric acid, quercetin, luteolin, apigenin, and isorhamnetin were found on *A. emarginatus* Labill caffeic by UHPLC-DAD-HRMS [67]. Secondary plant metabolites known as polyphenols are typically involved in defense against pathogens or UV radiation. The high phenolic content in plant extracts indicates the high antioxidant capacity of the plant [68]. Epidemiological studies report that polyphenols protect against the onset of cancer, cardiovascular disease, diabetes, osteoporosis, and neurological diseases [69].

Compared with these studies, our study’s sample (*A. alopecurus*) showed differences in phenolic compounds found by LC-MS/MS analysis. The differences in observed phenolic compounds can be attributed to many factors, such as age, variety difference, growing medium, method of harvesting, and more. However, this is consistent with the literature, as fumaric acid, isorhamnetin, chlorogenic acid, rosmarinic acid, and other phenolics make up a significant part of the phenolic compounds found within Astragalus species. This study provided the first important finding regarding the high concentration of isorhamnetin, which has extensive pharmacological activities, including cardiovascular and cerebrovascular protection, anti-tumor, anti-inflammatory, anti-oxidation, organ protection, prevention of obesity, etc. [67]. Other findings were the presence of high levels of fumaric acid, which is used as a food additive and as a food acidity regulator due to its antimicrobial, anti-tyrosinase, and antioxidant properties, and high levels of rosmarinic acid, which is used to extend the shelf life and improve the quality of foods [70].

In a study, the phenolic contents of the aerial part and root extract of *A. dumanii* were detected as 13.23 and 5.31 mg GAE/g, respectively, and the flavonoid contents were found to be 7.93 and 8.26 mg QE/g [17], respectively. The total phenolic and flavonoid contents in *A. brachycalyx* ethanol extracts were found to be 23.182 μg GAE/mg and 4.672 μg QE/mg, respectively [16]. The total phenolic and flavonoid contents in the various extracts of *A. lagurus* were found to be 20.34–20.72 mg GAEs/g and 19.58–31.10 mg REs/g, respectively [71]. In a study, the total phenolics and flavonoids in the methanol extract of *A. squarrosus* were found to be 23.3 mg GAEs/g and 26.0 mg QE/g, respectively [72]. In another study, the total phenolics and flavonoids in the methanol extracts of the stem parts of *A. diphtherites* and *A. gymnalopecias* were found to be 76.1 ± 0.9, 54.66 ± 2.3 μg GAE/mg and 39.31 ± 0.2, 36.81 ± 0.3 μg QE/mg, respectively. The following results were reported in the study conducted by Albayrak and Kaya [73], in which the phenolic content and antioxidant activity of four Astragalus species (*A. gummifer*, *A. microcephalus*, *A. talasseu*, and *A. acmophyllus*) were determined: The yields of the plants are between 9.78 and 16.38. Total phenolic contents are between 5.49 and 13.49 mg GAE/g extract. Total flavonoid contents are between 0.76 and 2.19 mg QE/g extract. DPPH IC_50_ values are between 86.67 and 253.88 μg/mL. When the percent inhibition values of the iron chelating activity of the extracts were measured at 5 mg/mL, the percent inhibition value of standard EDTA was 99.45%, while the extracts ranged from 43.88% to 68.35%. In addition, the amounts of chlorogenic acid, epicatechin, catechin hydrated, rutin, quercetin, kaempferol, syringic acid, cinnamic acid, and ferulic acid of Astragalus species were determined by LC-MS/MS. In the LC-MS/MS analysis of the extracts, it was reported that the main component was ferulic acid (1123.9 ppm), followed by syringic acid (735.18 ppm) and cinnamic acid (558 ppm), and the least abundant compound was quercetin (293.5 ppm) [73].

It was reported that methanol, ethyl acetate, butanol, and aqueous extracts made from the aerial parts of *A. bombycinus* had low DPPH free radical scavenging activity. Inhibition values at the dose of 0.1 g/mL were found to be 12.2, 11.5, 8.5, and 8.2 for the extracts, respectively [74]. The IC_50_ value for the *A. globosus* methanolic extract is 196.4. For the positive control, butylated hydroxytoluene, this value is 19.8 μg/mL. In the β-carotene/linoleic acid system, *A. globosus* methanolic extract has 35.9% activity, while hexane/dichloromethane extract has 48.7% activity [75]. The n-butanol extract of *A. monspessulanus*’ aerial parts was reported as 2.09 g/mL [76]. It has been reported that the IC_50_ values of DPPH radical scavenging activity of ethanol extracts obtained from the aerial and root parts of *A. dumanii* are 1398 and 1009 μg/mL, respectively. In addition, the IC_50_ values of ABTS radical scavenging activity were reported as 1.18 and 82.25 μg/mL, respectively [17].

In the study conducted by Albayrak and Kaya [73], the antioxidant activity of four Astragalus species was examined. Ferric ions (Fe^3+^) reducing the capacity of the extracts are between 0.60 and 4.25 mM/L. When the reducing power of ferric ions (Fe^2+^) is measured, the absorbance value of standard BHT is 2.249 and that of extracts is between 1.118 and 2.172. When cupric absorbance values are measured at 1 mg/mL, the absorbance value of standard Trolox is 2.85, while the extracts are between 0.32 and 0.83 [77]. In another study, it was reported that *A. lagurus* water extract had a reducing power of 73.98 mg TEs/g for CUPRAC and 53.49 mg TEs/g for FRAP [71].

Experimental studies support the use of natural products as a source of antioxidants against neurodegeneration [78]. According to the literature review, it was found that the AChE inhibition effects of ether extracts of *A. leporinus*, *A. distinctissimus*, and *A. schizopterus* plants were determined as follows: 46.96 ± 4.06, 54.71 ± 0.09, and 22.01 ± 0.07%, respectively. In the discovery of antidiabetic compounds with fewer side effects, in vitro experiments may be preferred, as in vivo experiments involve more expensive and ethical responsibilities. When the literature was searched, it was observed that *A. brachycalyx* ethanol extract showed IC_50_ values of 0.620 μg/mL on α-glycosidase, 0.306 μg/mL on α-amylase enzymes, and 1.985 μg/mL on AChE. Tacrine was used as a positive control for AChE inhibition with an IC_50_ value of 0.597 nM against AChE [16].

The three-dimensional structure of human hCA II has been demonstrated by X-ray crystallography [79]. It contains a zinc ion (Zn^2+^) along with residues Thr199, Glu106, and His64 that directly participate in the catalytic activity in the active site of this dominant and cytosolic isozyme. The His94 residue acts as a shuttle, transferring a proton from the zinc-bound water to the solvent medium. MEAA and WEAA were tested against cytosolic hCA II isoenzymes. According to Table 4, it is depicted that extracts inhibit the hCA II enzyme. hCA isozymes take part in some biochemical and physiological processes as well as playing an important role in some diseases, such as cerebral edema, obesity, cancer, glaucoma, altitude sickness, and epilepsy. Cytosolic hCA II is highly expressed in most organs and contributes to many important physiological processes. Recently, hCA inhibitors have been commonly used as novel antiglaucoma, diuretics, antiobesity, anticancer, anticonvulsant, and anti-infective medications [65].

In addition to metabolically providing CO_2_ transfer, the hCA enzyme plays a role in the accumulation of H^+^ and HCO_3_^−^ in many tissues. The hCA II isoenzyme is one of the most effective enzymes in erythrocytes and is found in almost every tissue and organ, including the eye, cornea, ciliary epithelium, kidney, central nervous system, and inner ear [80]. It was claimed that hCA II causes glaucoma and impaired vision by increasing HCO_3_^−^ secretion in the eye’s anterior uvea. They are involved in the secretion of bicarbonate from the exocrine glands in the digestive system [81]. They play a role in regulating the acidity of gastric juice and in the secretion of mucus and bicarbonate from epithelial cells on tissue surfaces in the gastrointestinal tract. In addition, the hCA II isoenzyme is effective in adjusting the intracellular pH and Ca^2+^ level to prevent bone resorption [82]. Most hCA I is found in erythrocytes. However, its activity is only 15% of the hCA II isoform. Of all the CAs, CA II has the largest cytosolic distribution and is an isozyme with high activity [83]. Many phenolic acids and phenolic natural products, such as p-hydroxybenzoic acid, p-coumaric acid, ellagic acid, caffeic acid, ferulic acid, gallic acid, tannic acid, syringic acid, quercetin, ellagic acid, etc., have the ability to inhibit carbonic anhydrase. To the best of our knowledge, carbonic anhydrase enzyme inhibition has been tested for the first time on Astragalus species. Thus, the gap in the literature has been filled.

## 4. Materials and Methods

### 4.1. Chemicals 

The phenolic compounds used in the LC-MS/MS analysis as standards (quinic acid, fumaric acid, gallic acid, pyrogallol, keracyanin chloride, cyanidin-3-*O*-glucoside, chlorogenic acid, catechin, peonidin-3-*O*-glucoside, 4-hydroxy benzoic acid, epicatechin, epigallocatechin gallate, caffeic acid, vanillic acid, syringic acid, vitexin, naringin, ellagic acid, hesperidin, p-coumaric acid, sinapic acid, taxifolin, ferulic acid, rosmarinic acid, vanillin, myricetin, resveratrol, luteolin, quercetin, apigenin, naringenin, isorhamnetin, chrysin, galangin, and curcumin) were obtained from Sigma-Aldrich (Steinheim, Germany). Formic acid and HPLC-grade methanol used to prepare calibration solutions and mobile phases were procured from Sigma-Aldrich (Steinheim, Germany). Analytical-reagent-grade methanol was obtained from Riedel-de Haen (Seelze, Germany). All other solvents and chemicals used in this study were of analytical grade. Milli-Q-grade water was obtained with the Milli-Q system; EASY pure RF. DMPD, DPPH, ABTS, standards, enzymes, and substrates were obtained from Sigma-Aldrich GmbH (Steinheim, Germany).

### 4.2. Plant Material

The aerial parts of *A. alopecurus* were collected by Dr. Leyla Güven from Erzurum Köşk village, 40°6′13″ K, 41°24′32″ D, at 1890 m altitude, on 1 July 2018. *A. alopecurus* was diagnosed by Prof. Dr. Yusuf Kaya from the Atatürk University Science Faculty. The herbarium specimens have been conserved at the Biodiversity Application and Research Center of Atatürk University with the AUEF 1395 herbarium number.

### 4.3. Preparation of Extracts (MEAA and WEAA)

For the preparation of MEAA, 50 g of aerial parts of the plant were crushed and extracted with 500 mL of methanol at room temperature using a mechanical stirrer [84]. The resulting mixture was filtered through Whatman No. 1 paper and concentrated until the rotary evaporator (Heidolph VV2000, Schwabach, Germany) was completely free of methanol. The resulting extract was stored at −18 °C until the study was carried out [85].

To prepare WEAA of the above-ground part of the plant, 500 mL of boiled water was poured onto the 30 g powdered plant, stirred in a magnetic stirrer for 1 h, and filtered with Whatman No. 1 paper [86]. The extract was evaporated in a rotary evaporator (Heidolph VV2000 Schwabach, Germany) and lyophilized. The lyophilized extract was stored at −18 °C until the study was carried out. The yields of methanol and water extracts from the aerial parts of the plant are 23.64 and 20.60% (*w/w*), respectively [87].

### 4.4. Determination of Total Phenolic and Flavonoid Contents

The total phenol content in MEAA and WEAA was determined by a spectrophotometric method based on the color reaction of phenolic compounds with the Folin-Ciocalteu reagent [88], as described in a previous study [89]. A spectrophotometric method based on the color reaction of flavonoids with aluminum chloride and potassium acetate was used to determine the total flavonoid content in plant extracts [90]. The quantity of total phenolics and flavonoids in extracts of *A. alopecurus* was determined as gallic acid equivalent (GAE) and quercetin equivalent (QE) from the equations obtained from the graphics of standards [91].

### 4.5. LC-MS/MS Instrumentation and Chromatographic Conditions

The phenolic composition in MEAA was detected by an Agilent Technologies 1290 Infinity UPLC chromatography equipped with an Agilent 6460 Triple Quadrupole mass spectrometer (Agilent Technologies, Palo Alto, Santa Clara, CA, USA) equipped with an electrospray ionization (ESI) source operating in negative multiple reaction monitoring (MRM) modes [92]. Chromatographic separation was performed on a Zorbax SB-C18 (4.6 × 100 mm, 3.5 µm) column at 30 °C by using a mobile phase consisting of water containing 0.1% formic acid (A) and acetonitrile containing 0.1% formic acid (B). The chromatography was performed by gradient elution. The gradient profile (time, % B) set was as follows: 0–4 min, 5% B; 4–7 min, 20% B; 7–14 min, 90% B; 15 min, 90% B; 15–15.1 min, 5% B; 15.1–17 min, 5% B at a 0.4 mL/min flow rate. An aliquot (5 μL) of the sample was injected, and the total run time was 17 min. The mass spectrometry conditions were set with a nitrogen gas temperature of 350 °C with a flow rate of 12 L/min, a sheath gas temperature of 250 °C with a flow rate of 5 L/min, and a nebulizer gas pressure of 55 psi. A complete mass scan ranging from 50 to 1300 *m/z* and the Agilent MassHunter Workstation to complete data acquisition and analysis were used.

### 4.6. Fe^3+^ Reducing Assay

The reducing capacity of MEAA and WEAA was assessed using the Fe^3+^ reducing technique, which differed from the FRAP and CUPRAC procedures [93], as given in the details [94]. The direct reduction in Fe^3+^(CN^−^)_6_ identified the decreasing quantity in this manner. Then, by adding excess ferric ions (Fe^3+^), the Perls’ Prussian blue complex was formed [95]. First, 0.75 mL of MEAA and WEAA with varying concentrations (15–45 g/mL) were mixed with K_3_Fe(CN)_6_ (1%, 1.25 mL) and buffer (1.25 mL, 0.2 M, pH 6.6) solutions. The mixture was then incubated for 30 min at 50 °C. Next, the mixture was treated with 1.25 mL of trichloroacetic acid (TCA, 10%) and 0.5 mL of FeCl_3_ (0.1%) before the absorbance was measured at 700 nm [96].

### 4.7. FRAP Reducing Assay

The FRAP approach is based on the acidic reduction in the Fe^3+^-TPTZ combination. At 593 nm, the enhanced absorbance was detected [97], as given in prior studies [98]. A fresh TPTZ solution (10 mM) was made and combined with buffer solution (pH 3.6, 0.3 M) and a 20 mM FeCl_3_ solution in water for this purpose. Different amounts of MEAA and WEAA (15–45 μg/mL) were dissolved in 5 mL of suitable buffer, mixed, and kept at 25 °C for 30 min. Finally, absorbances at 593 nm were measured [99].

### 4.8. Cu^2+^ Reducing Assay

The CUPRAC test was used to assess the reducing capabilities of MEAA and WEAA [100], as given in a prior study [101]. Neocuproine was utilized as a chromogenic oxidizing agent in this approach [102]. To begin, 1 mL of acetate buffer (1.0 M), neocuproine (7.5 mM), and CuCl_2_ solution (10 mM) were added to each tube and vortexed. All samples were put into tubes at concentrations ranging from 15 to 45 μg/mL. With distilled water, the tubes were filled to 1 mL. The samples were maintained at 25 °C for 30 min, and the absorbance was recorded at 450 nm [103].

### 4.9. DPPH Radical Scavenging Assay

Using the DPPH scavenging technique, the free radical scavenging capacity of MEAA and WEAA was assessed [103]. The technique relies on antioxidants to remove DPPH free radicals. Standards and extracts were generated with concentrations ranging from 15 to 45 μg/mL. For each sample, 500 μL of DPPH (0.1 mM) was added to tubes. For 30 min, these tubes were kept in the dark at 25 °C. At 517 nm, the measurements were taken. Samples of DPPH potentials were calculated and compared to standards. Finally, the IC_50_ values for each sample were determined. The decrease in absorbance demonstrates the sample’s capacity to scavenge DPPH free radicals [104].

### 4.10. ABTS Radical Scavenging Assay

A second radical scavenging technique, the ABTS^•+^ scavenging test [104], was employed to assess MEAA and WEAA’s capacity to scavenge free radicals [105]. First, an ABTS radical cation was produced, per this experiment, and then K_2_S_2_O_8_ (2.45 mM) and ABTS (7.0 mM) interacted [106]. Prior to measurement, the solution’s absorbance was corrected with buffer solution to 0.750 ± 0.025 at 734 nm (pH 7.4, 0.1 M). Then, MEAA and WEAA at varying concentrations (15–45 μg/mL) were added to 1 mL of ABTS^•+^ solution. ABTS^•+^ scavenging of all samples was assessed at 734 nm after 30 min. The decrease in absorbance demonstrates the sample’s capacity for free radical scavenging when treated with ABTS^•+^ [107].

### 4.11. DMPD Radical Scavenging Assay

The DMPD^•+^ scavenging potential of MEAA and WEAA was determined using a slightly modified approach described previously by Fogliano et al. [108] and a prior study [109]. For this purpose, 200 µL of 0.05 M FeCl_3_ and 1 mL of DMPD solution were added to the 0.1 M buffer (100 mL, pH 5.3). All samples were produced with concentrations ranging from 15 to 45 μg/mL. Water was used to reduce the total volume to 0.5 mL. After an hour of incubation, 1 mL of DMPD^•+^ solution was transferred, and absorbance at 505 nm was measured prior to the studies [110].

### 4.12. Metal Chelating Assay

The Fe^2+^ chelating effect of MEAA and WEAA was carried out as described in the literature by Re et al. [111] and previous studies [112]. Different concentrations (15–45 μg/mL) of extracts and standard compounds were transferred to a 0.125 mL FeSO_4_ (2 mM) solution. In this way, Fe^2+^ ions interact with the phenolic compounds in the extract, and Fe^2+^ ions are chelated by the sample. Next, 0.5 mL of Tris-HCl solution (pH 7.4) is added and incubated for 30 min. Then, 0.75 mL of 0.2% bipyridyl solution dissolved in HCl (0.2 M), 0.125 mL of ethanol, and 0.595 mL of water are added to the mixture, respectively, and incubated for 15 min. Ethanol was used as a blank, and absorbances were measured at 522 nm [113].

### 4.13. Anticholinergic Assay

The inhibition properties of MEAA and WEAA against AChE from *Electrophorus electricus* were examined according to previous studies [114,115]. In this study, 100 µL of Tris/HCl buffer (pH 8.0, 1 M), 50 µL of 5.5′-dithiobis (2-nitrobenzoic acid) (DTNB) (0.5 mM), 5–20 µL of AChE solution (5.32 × 10^−3^ EU), 10–100 µL of sample solutions, and 50 µL of acetylcholine iodate (1 µmol) used as substrate were mixed. It was incubated for 15 min at room temperature for the reaction to occur. Finally, the activity of the mixture was measured at 412 nm [116].

### 4.14. Antidiabetic Assay

The ability of methanol and water extracts to inhibit α-glycosidase and α-amylase enzymes was examined to establish the plant’s potential as an antidiabetic. A p-Nitrophenyl-*D*-glycopyranoside (p-NPG) substrate, as described in a previous study [117], was used to test the inhibitory effectiveness of MEAA and WEAA on α-glycosidase and α-amylase enzymes. At first, α-glycosidase enzyme solution (10–30 µL, 0.15 U/mL), the sample (10–50 µL), and 50 µL of p-NPG (5 mM) were mixed and incubated for 3 min at room temperature. The absorbance of the mixture was monitored at 405 nm prior to the study [118].

For the α-amylase enzyme inhibition assay, it was performed according to our previous study [119]. First, 2 g of starch was dissolved in a 100 mL solution of 0.4 M NaOH before being heated at 80 °C for 15 min. The pH was changed to 6.9 after cooling, and distilled water was used to make the total volume 100 mL. Then, 5 μL of extracts were added to 35 μL of phosphate buffer (pH 6.9) and 35 μL of starch solution. Next, 20 μL of the enzyme solution was added, and the mixture was again incubated at 25 °C for 20 min. The reaction was finished by adding 50 μL of HCl (0.1 M). At a wavelength of 580 nm, the mixture’s absorbance was measured prior to the studies [120].

### 4.15. Antiglaucoma Assay

In order to examine the inhibitory effects of methanol and water extracts of *A. alopecurus* aerial parts on the hCA II isoform, this isoform was purified by Sepharose-4B-L-Tyrosine-sulfanilamide affinity chromatography from human red blood cells [121]. Human erythrocyte samples were centrifuged for 30 min at 13,000 rpm for this reason. After that, the solution was filtered. At pH 8.7, solid Tris was added to the serum to isolate the hCA II isoenzyme. With buffer solution (pH 8.7, 25 mM Tris-HCl/0.1 M Na_2_SO_4_), the affinity column was adjusted. The hCA II isoenzyme was cleaned with the buffer solution (pH 5.6, 0.1 M sodium acetate/0.5 M NaClO_4_). The hCA II isoenzyme was fractionated from the column into Eppendorf tubes (2 mL). All investigations were conducted at 4°C. A Thermo Scientific brand spectrophotometer was used to monitor the change in absorbance of the p-nitrophenolate ion of p-nitrophenylacetate for 3 min at room temperature in order to determine the activity of the hCA II isoenzyme [122]. The measured test tube has the following contents: 0.1 mL of enzyme solution, 0.2 mL of water, and 0.4 mL of 0.05 M Tris-SO_4_ buffer (pH 7.4) [123]. The purity of the hCA isoform was controlled by the SDS-PAGE purity technique [124]. The protein quantity was determined at 595 nm according to the Bradford method [125], as given previously [126]. The spectrophotometric Verpoorte method (Shimadzu, UVmini-1240 UV–VIS) was employed for performing CA activity [73,127].

### 4.16. IC_50_ Value Determination

The half maximal inhibition concentration (IC_50_) values were calculated from activity (%) versus different concentrations of MEAA and WEAA [128].

### 4.17. Statistical Analysis

Statistical analyses were used to evaluate anticholinergic, antidiabetic, and antioxidant activity results by unpaired Student’s *t*-test (GraphPad, La Jolla, CA, USA. Software 7.0). All results were given as means with their standard deviation (SD). *p* < 0.05 was taken as the minimum level of significance.

## 5. Conclusions

Our study provided important findings regarding the high concentration of isorhamnetin, fumaric acid, and rosmarinic acid in *A. alopecurus* with an LC-MS/MS analysis, and it was concluded that *A. alopecurus* can be used commercially due to its phenolic compounds, antioxidants, and enzyme inhibition abilities. *A. alopecurus* contains quantities of bioactive secondary metabolites, such as phenolic and flavonoid compounds. Furthermore, the *A. alopecurus* extract was found to be rich in phenolic contents, antioxidant ability, reducing power, AChE, α-amylase, α-glycosidase, and hCA II inhibition profiles. *A. alopecurus* can also be used as a natural source for the treatment of T2DM, AD, and glaucoma. From this perspective, inhibition studies on the AChE enzyme are planned to determine the anti-AD effects of *A. alopecurus* extracts. Additionally, the inhibition of the hCA II enzyme was analyzed to determine a link with glaucoma. Furthermore, the antidiabetic potential of *A. alopecurus* extracts has been realized by identifying α-amylase and α-glycosidase inhibition. Additionally, Cu^2+^, Fe^2+^, and Fe^3+^-TPTZ reducing, as well as DPPH and ABTS scavenging, tests were performed to understand the antioxidant ability of *A. alopecurus* extracts. Furthermore, total phenolics and flavonoids in *A. alopecurus* were determined for both extracts. A phenolic analysis was performed by LC-MS/MS to define the biological effects of the chemical profile of *A. alopecurus*. Although our current laboratory conditions are not suitable, we plan to conduct in vivo studies for this research in the future, which will be supported by experimental animals.

## Figures and Tables

**Table 1:** Quantitative screening of phenolic compounds in the methanolic extract (MEAA) of the aerial part of *A. alopecurus* by LC-MS/MS.

No.	Analytes	RT ^a^	M.I. (*m/z*) ^b^	F.I. (*m/z*) ^c^	Ion. Mode	MEAA (µg/mL)
1	Quinic acid	2.36	190.9	85.0	Neg	N.D.
2	Fumaric acid	3.94	114.9	71.1	Neg	855.07
3	Gallic acid	5.45	168.9	79.0	Neg	N.D.
4	Pyrogallol	6.53	124.9	96.6	Neg	N.D.
5	Keracyanin chloride	10.49	592.8	284.7	Neg	N.D.
6	Cyanidin-3-*O*-glycoside	10.53	447.1	283.8	Neg	N.D.
7	Chlorogenic acid	10.91	352.9	190.9	Neg	40.92
8	Catechin	10.93	289.1	244.9	Neg	N.D.
9	Peonidin-3-*O*-glucoside	10.98	460.9	298.8	Neg	N.D.
10	4-OH-Benzoic acid	11.27	137.0	93.1	Neg	57.23
11	Epicatechin	11.42	289.0	244.9	Neg	N.D.
12	Epigallocatechin gallate	11.52	456.8	304.9	Neg	N.D.
13	Caffeic acid	11.51	178.8	134.8	Neg	N.D.
14	Vanillic acid	11.76	166.9	151.9	Neg	N.D.
15	Syringic acid	11.80	169.9	122.8	Neg	N.D.
16	Vitexin	11.77	430.9	310.9	Neg	N.D.
17	Naringin	12.01	579.0	270.8	Neg	N.D.
18	Ellagic acid	12.09	300.8	283.4	Neg	48.54
19	Hesperidin	12.17	609.0	300.9	Neg	N.D.
20	p-Coumaric acid	12.27	163.0	118.9	Neg	32.26
21	Sinapic acid	12.56	222.8	163.9	Neg	N.D.
22	Taxifolin	12.38	302.9	124.7	Neg	N.D.
23	Ferulic acid	12.47	193.0	134.0	Neg	N.D.
24	Rosmarinic acid	12.53	358.8	160.8	Neg	64.54
25	Vanillin	12.60	151.0	135.8	Neg	N.D.
26	Myricetin	12.58	316.9	150.9	Neg	N.D.
27	Resveratrol	13.16	226.8	184.8	Neg	N.D.
28	Luteolin	13.35	284.9	132.9	Neg	5.50
29	Quercetin	13.54	300,9	150.7	Neg	10.88
30	Apigenin	13.96	268.9	224.8	Neg	N.D.
31	Naringenin	14.11	270.9	150.8	Neg	1.44
32	Isorhamnetin	14.13	314.9	299.8	Neg	1489.11
33	Chrysin	15.59	252.8	208.8	Neg	N.D.
34	Galangin	15.71	268.9	168.8	Neg	N.D.
35	Curcumin	16.29	366.9	148.9	Neg	N.D.

^a^ R.T.: Retention time. ^b^ MI (*m*/*z*): Molecular ions of the standard analytes (*m*/*z* ratio). ^c^ FI (*m*/*z*): Fragment ions. N.D.: Not detected.

**Table 2:** Ferrous ions (Fe^3+^), cupric ions (Cu^2+^), and ferrous ions (Fe^3+^)-2,3,5-triphenyltetrazolium chloride (TPTZ) ions reduction capabilities of methanolic (MEAA) and water (WEAA) extracts of the aerial part of *A. alopecurus* and positive controls at 30 μg/mL concentration.

Antioxidants	Fe^3+^ Reducing		Cu^2+^ Reducing		Fe^3+^-TPTZ Reducing	
	λ_700_	r^2^	λ_450_	r^2^	λ_593_	r^2^
BHA	1.257	0.9523	1.800	0.9742	0.884	0.9899
BHT	2.018	0.9466	2.912	0.9969	2.089	0.9581
*α*-Tocopherol	1.895	0.9402	1.139	0.9967	1.995	0.9807
Trolox	1.545	0.9966	2.323	0.9980	1.755	0.9990
MEAA	0.308	0.9971	0.163	0.9918	0.284	0.9742
WEAA	0.284	0.9910	0.137	0.9999	0.314	0.9894

**Table 3:** The half maximal inhibition concentration (IC_50_; μg/mL) of MEAA, WEAA, and standards for 1,1-diphenyl-2-picrylhydrazyl radicals (DPPH^•^), 2,2′-azino-bis(3-ethylbenzothiazoline-6-sulfonic acid) (ABTS^•+^), N,N-dimethyl-p-phenylene diamine (DMPD^•+^) scavenging, and ferrous ions (Fe^2+^) chelating effects.

Antioxidants	DPPH^•^ Scavenging		ABTS^•+^ Scavenging		DMPD^•+^ Scavenging		Fe^2+^ Chelating	
	IC_50_	r^2^	IC_50_	r^2^	IC_50_	r^2^	IC_50_	r^2^
BHA	9.00	0.9399	7.71	0.9330	31.43	0.9993	-	-
BHT	21.00	0.9668	7.71	0.9330	-	-	21.66	0.9908
Trolox	5.92	0.9770	7.71	0.9330	14.38	0.9349	-	-
α-Tocopherol	9.63	0.9947	8.10	0.9550	-	-	-	-
MEAA	99.02	0.9977	32.21	0.9987	231.05	0.9967	46.21	0.9717
WEAA	115.53	0.9934	30.22	0.9976	65.22	0.9987	33.01	0.9601

**Table 4:** The inhibition values of MEAA and WEAA against α-glycosidase, α-amylase, acetylcholinesterase (AChE), and carbonic anhydrase II (CA II) enzymes. Acetazolamide is a standard inhibitor of carbonic anhydrase II (CA II) isoenzyme inhibition. Tacrine is a positive control for acetylcholinesterase (AChE) inhibition. Acarbose is a positive control for α-glycosidase and α-amylase enzyme inhibition.

Inhibitors	α-Amylase		α-Glycosidase		AChE		hCA II	
	IC_50_	r^2^	IC_50_	r^2^	IC_50_	r^2^	IC_50_	r^2^
MEAA (µg/mL)	693.15	0.9647	9.07	0.9775	1.99	0.9923	147.70	0.9804
WEAA (µg/mL)	346.58	0.9387	2.24	0.9155	2.45	0.9930	171.70	0.9671
Acetazolamide (nM)	-	-	-	-	-	-	8.37	0.9825
Tacrine (nM)	-	-	-	-	0.0246	0.9706	-	-
Acarbose (nM)	6.46	0.9424	14.72	0.9922	-	-	-	-

## Data Availability

Data available in a publicly accessible repository.
